# In vivo and in vitro toxicity evaluation of liposome-encapsulated sirolimus

**DOI:** 10.1186/s40942-019-0186-7

**Published:** 2019-09-24

**Authors:** Murilo Batista Abud, Ricardo Noguera Louzada, David Leonardo Cruvinel Isaac, Leonardo Gomes Souza, Ricardo Gomes dos Reis, Eliana Martins Lima, Marcos Pereira de Ávila

**Affiliations:** 1 Federal University of Goias, Goiania, GO Brazil; 2 School of Pharmacy, Federal University of Goias, Goiania, GO Brazil; 3 Present Address: Instituto de Olhos São Sebastião, Largo do Machado 54, 1208, Rio de Janeiro, RJ 22221-020 Brazil

**Keywords:** Sirolimus, LES, ARPE-19, Rapamycin, Uveitis

## Abstract

**Background:**

To evaluate the in vivo and in vitro toxicity of a new formulation of liposome-encapsulated sirolimus (LES).

**Methods:**

In vitro experiments were done using ARPE-19 and HRP cells. An MTT assay was used to determine cell metabolic activity and a TUNEL assay for detecting DNA fragmentation. In vivo experiments were conducted on New Zealand albino rabbits that received intravitreal injections of empty liposomes (EL) or different concentrations of LES. Histopathological and immunohistochemical analyses were performed on the rabbit’s eyes following injection.

**Results:**

Eighteen eyes of nine rabbits were used. MTT assay cell viability was 95.04% in group 1 (12.5 µL/mL LES). 92.95% in group 2 (25 µL/mL LES), 91.59% in group 3 (50 µL/mL LES), 98.09% in group 4 (12.5 µL/mL EL), 95.20% on group 5 (50 µL/mL EL), 98.53% in group 6 (50 µL/mL EL), and 2.84% on group 8 (50 µL/mL DMSO). There was no statistically significant difference among groups 1 to 7 in cell viability (p = 1.0), but the comparison of all groups with group 8 was significant (p < 0.0001). The TUNEL assay comparing two groups was not statistically significant from groups 1 to 7 (p = 1.0). The difference between groups 1 to 7 and group 8 (p < 0.0001) was significant. Histopathological changes were not found in any group. No activation of Müller cells was detected.

**Conclusion:**

A novel formulation of LES delivered intravitreally did not cause in vitro toxicity, as evaluated by MTT and TUNEL assays, nor in vivo toxicity as evaluated by histopathology and immunohistochemistry in rabbit eyes.

## Background

Uveitis is a condition characterized by inflammation of the uveal tract. It is responsible for 5% to 20% of legal blindness cases in the United States and Europe, and up to 25% of cases in developing countries [1]. It can be classified according to its location in the eye: anterior (iritis and irodocyclitis), intermediate (ciliary body, extreme periphery of the retina and adjacent choroid), posterior (choroid and retina) and pan-uveitis (inflammation of all uvea) [2]. Uveitis onset is typically between the third and seventh decades of life, and therefore affects patients during their most active and economically productive years. As such, uveitis is responsible for a significant global contribution to visual impairment and reduction in quality of life [1, 3].

Uveitis can be infectious or non-infectious (NIU). It comprises several different ocular inflammatory conditions which may differ in presentation, clinical course, and response to therapy [4], but is often caused by autoimmune conditions, with several cases described as idiopathic [5, 6]. In the United States, 79% of NIU cases affect the intermediate, posterior or entire uvea, while 21% of cases are purely anterior and do not cause vitreous opacities [7]. Cellular activation of macrophages and T-lymphocytes is involved in the pathophysiology of non-infectious uveitis, culminating in tissue inflammation [8].

The first choice in the treatment of NIU are corticosteroids administered topically, orally, intravenously, periocularly, or intraocularly [9]. Corticosteroids reduce ocular inflammation and vitreous haze; however, chronic administration can lead to cataract formation, increased intraocular pressure, and induction of systemic conditions including diabetes mellitus, systemic arterial hypertension (SAH), and Cushing’s Syndrome [10].

Sirolimus, also known as rapamycin, is a macrolide antibiotic produced by organisms of the species *Streptomyces actinomycetohygroscopicus*. It was isolated from soil samples collected from the Rapa Nui region of Easter Island in the early 1970s as part of an effort to identify novel anti-microbial agents [11, 12]. Although it was initially discovered for its antibiotic properties, it was also found to have important immunosuppressive properties [13]. The immune actions of sirolimus are propagated through its target protein, mTOR (Mammalian Target of Rapamycin). mTOR is a kinase protein that regulates cell growth and proliferation in response to mitogens, growth factors, hormones, and nutrient availability [14]. Through this pathway, sirolimus acts as an immunomodulatory therapeutic (IMT) agent by suppressing T cell proliferation through inhibition of IL-2, 4, and 15 expression [13–15].

Sirolimus is currently approved for oral use as an immunosuppressant drug in the prevention of organ transplant rejection and as a therapeutic adjuvant following the placement of certain coronary stents [16, 17]. In addition to its immunosuppressive properties, it has been found to have anti-inflammatory, anti-fibrotic, anti-proliferative and anti-angiogenic activity, as well. As such, sirolimus has been considered as a possible agent in the treatment of retinal vascular disorders, which are characterized by pathologic proliferative and angiogenic processes [18].

Oral sirolimus was reported to be effective in the treatment of NIU; however, this application was limited by its multiple gastrointestinal and dermatological side effects [19]. Other studies have shown that systemic use of sirolimus was associated with a number of other cytotoxic effects, particularly hematological, which have limited its use in the management of uveitis [20–25].

The SAVE and SAVE-2 studies have demonstrated that intravitreal sirolimus appeared to be effective in reducing ocular inflammation in subjects with active or quiescent NIU [26, 27]. More recently, a phase III, multicenter randomized double-masked study, the SAKURA study, demonstrated that intravitreal sirolimus at a dose of 440 mg was associated with a significant improvement in ocular inflammation and vitreous opacification, as well as increased or preserved BCVA, in subjects with active posterior NIU. Minimal side effects were seen in this trial [28]. The SAVE and SAKURA trials have used a proprietary formula that increases the intravitreal half-life, which is otherwise limited by the low water solubility of sirolimus [26–28].

However, other potential formulations of sirolimus for ocular use remain an active area of investigation. Liposomes are spherical vesicles with at least one lipid bilayer, and are frequently used to entrap drugs for slow release [29, 30]. Previous studies have showed an increase in the half-life of intravitreal water-insoluble drugs, such as gentamicin, and decreased retinal toxicity of amphotericin B, when delivered in liposomal form [31, 32]. The purpose of this study is to evaluate the in vivo and in vitro toxicity of a novel liposome-encapsulated intravitreal formulation of sirolimus.

## Methods

### Liposome preparation

Sirolimus was purchased from LC Laboratories (Boston, MA, USA) and soy bean phosphatidylcholine (S100PC) from Avanti Polar Lipids (Alabaster, AL, USA). The liposomes were prepared using the thin lipid film hydration method followed by extrusion, as described previously [33–35]. The mixture of sirolimus (20 mg) and phosphatidylcholine (120 mg) was dissolved in chloroform and dried with nitrogen gas to form a thin lipid film on a round bottom glass tube. The tube was maintained under vacuum for 12 h to ensure complete removal of chloroform. The resulting film was hydrated with 4 mL of TES buffer containing 6% trehalose (pH 7.0) for self-assembly of the lipids into multilamellar liposomes. The liposome dispersion was then passed through 0.1 and 0.2 μm pore size polycarbonate membranes in an extruder (Northern Lipids, Inc., Burnaby, Canada) under nitrogen pressure, and was freeze–dried and stored at 4 °C.

### Entrapment efficiency of sirolimus in liposomes

Entrapment efficiency (EE) is defined as either the ratio of liposome-encapsulated drug to the total drug added to the liposomal dispersion, or as the molar ratio of the lipid to the drug. In order to determine EE, free, or non-encapsulated, sirolimus was removed from the liposomal suspensions by centrifuging at 3000 RPM for 15 min. 100 µL of the liposome supernatant was then dissolved in 1 mL of methanol. The sirolimus concentration was determined by high performance liquid chromatography (HPLC), and the EE and molar ratio (lipid:drug) determined from three individually prepared batches of liposome suspensions. The mean particle size and particle size distributions of the liposomes were determined using the Zeta Sizer Nano S (Malvern Instruments LTD, Worcestershire, UK). Readings were obtained following dilution of the liposomes with TES buffer.

### In vitro drug release studies

In vitro release of sirolimus from liposomes was investigated using dialysis. One milliliter of liposome suspension was placed into a dialysis tube (MWCO 6000–8000 Da) and tightly sealed. The dialysis tube was immersed in 100 mL of release medium (TES buffer pH 7.4 with 2% de sodium lauryl sulfate) and incubated in an orbital shaker for 4 days at 37 °C. At various time intervals the drug content in the release medium was analyzed by HPLC. Sink conditions were maintained throughout the release studies.

### Stability of sirolimus lyophilized liposome formulation

The stability of sirolimus containing lyophilized liposome formulation was evaluated in a kiln at 25 °C with 60% relative humidity for a period of 3 months. The study was performed only for the formulation with the highest drug concentration.

### In vitro toxicity assessment

The in vitro experiments used human RPE cells (ARPE-19) (American Type Culture Collections, Manassas, VA, USA), cultured as described previously by Dunn et al. [36] This cell line is not transformed and has structural and functional properties characteristic of RPE cells in vivo. The MTT assay was used to determine cell metabolic activity and the TUNEL assay for detecting DNA fragmentation related to apoptosis.

The RPE cells were suspended in the culture medium mentioned above and distributed at the concentration of 2 × 10^4^ in 200 µL on a 96-well plate. They were incubated for a period of 5 days until reaching confluence at 37 °C and 5% CO_2_, at which point the cell medium was removed and replaced with fresh medium of serum-free fetal bovine. For the MTT assay and TUNEL assays, two equal RPE cell experimentation series were prepared and one series used for each test exclusively. The eight groups prepared included three with liposome-encapsulated sirolimus at different concentrations (group 1: 62.5 µg/mL, group 2: 125 µg/mL, group 3: 250 µg/mL), three with empty liposomes at different concentrations (group 4: 62.5 µg/mL, group 5: 125 µg/mL, group 6: 250 µg/mL), a control group with 50 µL basic salt solution (BSS) (group 7), and a positive control group with 50 µL dimethyl sulfoxide (DMSO) (group 8). The desired vitreous concentration in human eyes was thus simulated. The experiment was carried for each of the six doses. After the addition of drugs, the cells were again incubated for 24 h. The medium was then removed and a new medium added with MTT 0.5 mg/mL (Sigma-Aldrich, Inc., St. Louis, MI, USA) in each well. The cells were incubated again for 4 h. Following this, the MTT medium was removed and 200 µL of isopropanol (Sigma-Aldrich, Inc., St. Louis, MI, USA) was added to extract formazan. The plates were shaken for 5 min and the absorbance corresponding to each sample measured on the Enzyme-Linked Immunosorbent Assay (ELISA) at 560 nm. The absorbance obtained from the control cells, treated with BSS, was considered as 100% cell viability [37].

For the TUNEL assay, the In Situ Cell Death Detection Fluorescein kit (Roche Diagnostics, Mannheim, Germany) was used. This kit consists of an enzyme solution and a marker solution. After 24 h of incubation, the cell media was completely removed and the cells fixed for 1 h in 4% paraformaldehyde solution at room temperature. The cells were then washed three times with PBS for 5 min, and were incubated in permeabilizing solution for 2 min at a temperature of 4° to 8 °C. The enzyme solutions (50 µL) and markers (450 µL) were mixed, obtaining 500 µL of the solution for detection of cell death by TUNEL (TUNEL solution). After the permeabilizing solution was removed, the cells were washed with PBS, and 50 µL of the TUNEL solution was added. The cells were incubated for 1 h at 37 °C in a humidified chamber protected from light. After removal from the TUNEL solution, the cells were washed with PBS and analyzed on an Olympus CKX41SF5 fluorescent microscope (Olympus Corporation, Center Valley, PA, USA) to count and quantify the total number of cells as well as the number of apoptosis-killed cells.

### Human retinal progenitor cell culture and TUNEL test

Human retinal progenitor cells (HRPCs) were cultured as described by KLASSEN et al. 2004.

For the TUNEL test, the In Situ Cell Death Detection Fluorescein kit (Roche, Mannheim, Germany), consisting of an enzyme solution and a marker solution, was used. HRPCs were cultured in cell culture flasks as described above. After adherence and on the verge of confluence, they were released from the flask with 0.25% trypsin (Sigma-Aldrich Inc. St. Louis, MO—United States) for 5 min at 37 °C in a humidified incubator. After 5 min in the incubator, the solution was removed from the culture flasks and centrifuged for 5 min at 1000 rpm. The supernatant was aspirated and cells were resuspended in culture medium for hRPCs. HRPCs were counted and plated in fibronectin coated 96-well plates at a density of 5000 cells/cm^2^.

After 48 h of cell culture, the cell medium was completely removed and new medium with different doses of liposome-encapsulated sirolimus, BSS and DMSO alone, was added. The groups were divided as follows: (A) Different doses of liposome-encapsulated sirolimus: Group 1: 62.5 μg/mL; Group 2: 125 μg/mL; Group 3: 250 μg/mL, (B) liposomes alone: Group 4: 62.5 μg/mL; Group 5: 125 μg/mL; Group 6: 250 μg/mL; (C) BSS/Control: Group 7: 50 µL. (D) DMSO—Positive Control: Group 8: 50 µL. For each group, the procedure was repeated in sextuplicate. After the respective drugs were added, the cells were kept for 24 h in a 37 °C incubator. After 24 h the cell medium was completely removed and the cells fixed for 1 h in 4% paraformaldehyde solution at room temperature. The cells were then washed with PBS for 5 min, repeated three times. Cells were incubated in a permeabilizing solution for 2 min at 4 °C to 8 °C. Enzyme (50 µL) and marker (450 µL) solutions were mixed to obtain 500 µL of TUNEL cell death detection solution (TUNEL solution). After the permeabilizing solution was removed, the cells were washed with PBS and 50 microliters of TUNEL solution was added. They were incubated for 1 h at 37 °C in a humidified chamber protected from light. Removing the TUNEL solution, the cells were washed with PBS and analyzed under an Olympus CKX41SF5 fluorescent microscope. The result of cells killed by apoptosis was quantified as a percentage. Cell nuclei from each well were counted and the result was divided by the number of cells marked for apoptosis killing and multiplied by 100.

### In vivo toxicity assessment

Nine New Zealand albino rabbits received intravitreal injections. All animals were treated according to the ARVO Statement for the Use of Animals in Ophthalmic and Vision Research. This research protocol was previously approved by the animal research ethics committee of the Federal University of Goias.

The animals were anesthetized by a veterinary anesthesiologist using a combination of 3 mg/kg of 2% xylazine hydrochloride (Calmiun, Uniao Quimica, Sao Paulo, Brazil) and 25 mg/kg ketamine (Ketamin, Cristalia, Sao Paulo, Brazil) administered intramuscularly. During anesthesia, the animals underwent oxygen therapy with 100% oxygen and an identification plate was implanted in one ear.

Before anesthesia, one drop of tropicamide 1% (Mydriacyl, Alcon Labs, Sao Paulo, Brazil) and one drop of 10% phenylephrine (Fenilfrina, Allergan, Sao Paulo, Brazil) were applied into each eye for pupil dilation. An ophthalmologic examination of the anterior and posterior segments of the ocular globe was performed using biomicroscopy and indirect binocular ophthalmoscopy, respectively. This examination was performed before the intravitreal injection and was repeated immediately after intravitreal injection and after 30 days.

The nine rabbits included in the study were randomly divided into two groups. The first group consisted of six rabbits, which received 250 µg of liposome-encapsulated sirolimus in the right eye. The left eye received empty liposomes alone (n = 3) or BSS as placebo (n = 3). The second group consisted of three rabbits, which received 500 µg of liposome-encapsulated sirolimus in the right eye (n = 3) and empty liposomes in the left (n = 3).

Intravitreal injection was performed 2 mm posterior to the limbus after instillation of 5% povidone iodine eye drops and 0.5% moxifloxacin antibiotic eye drops (Vigamox, Alcon Labs, Sao Paulo, Brazil). Immediately after intravitreal injection, each eye was evaluated through indirect binocular ophthalmoscopy to determine central retinal artery perfusion and possible complications related to the procedure, such as crystalline lens touch or retinal detachment. Thirty days after intravitreal injections, the rabbits were again anesthetized, and biomicroscopy of the anterior segment and fundoscopy with indirect binocular ophthalmoscopy were performed, followed by euthanasia and enucleation.

For euthanasia, the animals were given a high level of sedation (2% xylazine hydrochloride) and received an intravenous solution of 50 mg/kg 2.5% thiopental sodium, followed by an injection of 10% potassium chloride. Euthanasia was confirmed after the absence of heart beats and respiratory movements.

### Anatomopathological analysis

Hematoxylin and eosin-stained sections of the eye were examined on the Olympus Optical Microscope (model CKX41SF5) at 20× magnification (Olympus Corporation, Center Valley, PA, USA). The slides were photographed with a digital camera coupled to the microscope and evaluated by an ophthalmic-experienced pathologist.

For immunohistochemistry analysis, the eyes were first fixed in a solution of 10% buffered formalin. They were then submitted to razor cutting in order to remove a circular medial slice of approximately 3 mm in thickness, from the cornea to the optic nerve. The small tissue strips were washed three times for 10 min with PBS buffer and placed in a solution of acrylamide (AES) for 12 h to promote infiltration. A 10 mL solution of AES with 50 µL of 10% ammonium persulfate (Sigma-Aldrich, Inc., St. Louis, MO, USA) was prepared in a 15 mL test tube. The tissues were removed from the acrylamide solution and placed in the solution prepared in a refrigerated environment (4–8 °C) for polymerization for 1 h. After polymerization, the tissues were carefully cut into small blocks with a razor. The blocks were wrapped in Tissue Tek OCT cryoprotectant (Sakura Finetek, Inc., Torrance, CA, USA) and placed at a temperature of − 80 °C. Cuttings of approximately 6 µm thick were then made in a Cryostat (Leica Biosystems, Inc., Buffalo Grove, IL, USA) with the blades stored at − 20 °C. The slides were removed at room temperature and washed three times for 5 min with PBS. A blocking solution was prepared with 10% goat serum (GS) (Sigma-Aldrich, Inc., St Louis, MO, USA), 3% bovine serum albumin (BSA) (Sigma-Aldrich, Inc., St Louis, MO, USA) and 0.01% Triton-X (Sigma-Aldrich, Inc., St Louis, MO, USA). The slides were exposed to the blocking solution at room temperature for 1 h. Subsequently, they were washed with PBS for 5 min and incubated with anti-glial monoclonal antibody to the acidic protein (GFAP) (Sigma-Aldrich, Inc., St Louis, MO, USA) for 12 h in a humid chamber at 4 to 8 °C. After 12 h, the slides were washed with PBS solution and incubated for one hour with CY3 secondary antibody (Sigma-Aldrich, Inc., St. Louis, MO, USA) while protected from light. The slides were then immediately washed again with PBS, incubated with 0.5 µg/mL nuclear labels (DAPI) for 5 min, and washed once more with PBS. The material was then analyzed using the Olympus Optical Microscope (model CKX41SF5, Olympus Corporation, Center Valley, PA, USA).

### Statistical analysis

For the purposes of statistical analysis, p-values of ≤ 0.05 were considered statistically significant. For analysis of the MTT and TUNEL assays, the K–S test was used after verifying that the data was normally distributed. ANOVA post hoc tests, with Bonferroni’s correction for multiple comparisons, were then used. Fisher’s exact tests were applied to calculate the p-values for the immunohistochemical tests from the GFAP and histological analysis. There were no a statistically significant differences between all groups.

## Results

### Release studies in vitro of liposomes encapsulated with sirolimus

**Fig. 1:**
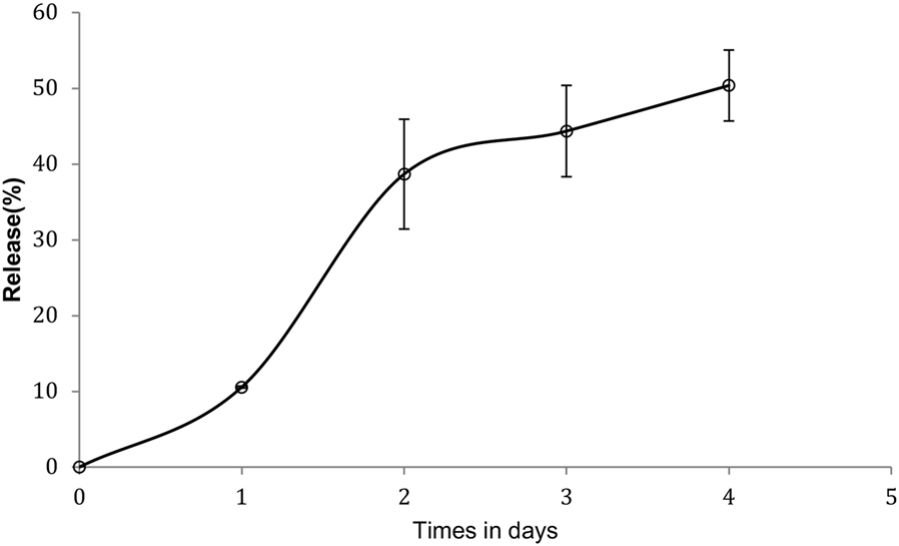
Release profile of sirolimus from liposomes

Mean diameter of sirolimus liposomes was 99.37 nm (± 11.90) with polydispersity index of 0.07 (± 0.01). Entrapment efficiency was 95.41% (± 4.27) (n = 3). The molar ratio was 1:7.7 (drug:lipid). In this sirolimus concentration there were no crystals of sirolimus. Rouf et al. obtained sirolimus liposomes with molar ration 1:50 (drug:total lipids) and particle size around 160 nm. Sirolimus liposomes obtained in this work showed better encapsulation efficiencies and a lower average diameter. In vitro release of rapamycin from liposomes occurred in a very slow pattern, indicating an efficient controlled release. After 4 days of the assay, the percentage of drug released was around 50%. Figure 1 show the release profile of sirolimus from liposomes. Rouf et al. studied the release of sirolimus from liposomes in the period of 24 h, and observed a drug release of 10% at this period.

### Stability results of sirolimus lyophilized liposome formulation

**Fig. 2:**
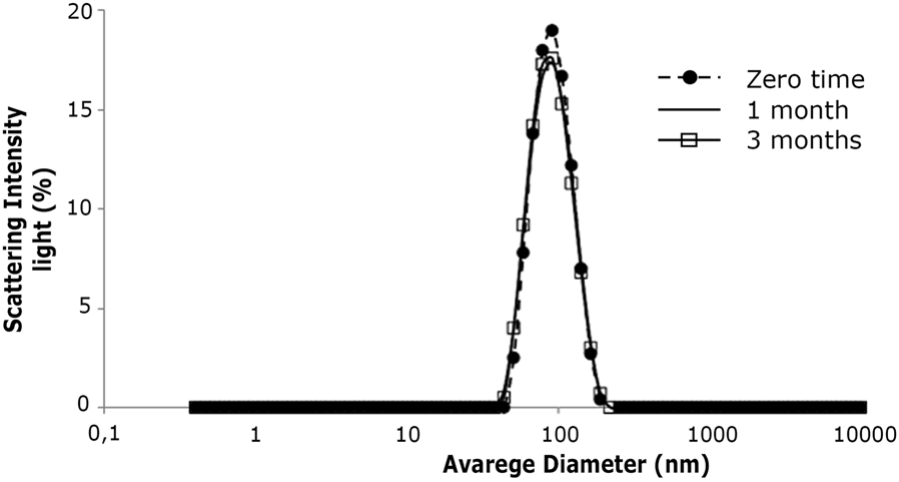
Average diameter of sirolimus containing lyophilized liposome formulation stored at 25 °C with 60% relative humidity and resuspended after 1 or 3 months of lyophilization

The sirolimus containing lyophilized liposome formulation was stable for at least 3 months under 25 °C with 60% relative humidity, with no changes in parameters of size, PDI and encapsulation efficiency (Table 1 and Fig. 2). No drug degradation occurred.

**Table 1:** Stability of sirolimus containing lyophilized liposomes at 25 °C with 60% relative humidity

Time (months)	Encapsulation efficiency
0	100*
1	98.87 ± 1.7
3	97.48 ± 2.9

* p > 0.05 when compared with 1 and 3 months

### MTT assay for ARPE19 cells

As mentioned previously, group 7 (BSS) was considered a control group with 100% viable cells for comparison to the other groups. The cell viability for each group is listed in Table 2. No statistical difference was detected between any of the individual groups and the control (group 7) cell viability (p = 1.0). There was also no statistically significant difference in pairwise comparisons between groups 1 to 7 in cell viability (p = 1.0). There was a significant difference in cell viability between each individual groups and the positive control group (group 8) (all p-values < 0.0001).

**Table 2:** ARPE-19 MTT assay results

Group	Medication	Cell viability (%)
1	12.5 µL/mL liposome-encapsulated sirolimus	95.04
2	25 µL/mL liposome-encapsulated sirolimus	92.95
3	50 µL/mL liposome-encapsulated sirolimus	91.59
4	12.5 µL/mL empty liposomes	98.09
5	25 µL/mL empty liposomes	95.20
6	50 µL/mL empty liposomes	98.53
7	50 µL/mL BSS	100.00
8	50 µL/mL DMSO	2.84

*BSS* balanced salt solution, *DMSO* dimethyl sulfoxide

### TUNEL assay for ARPE19 cells

**Fig. 3:**
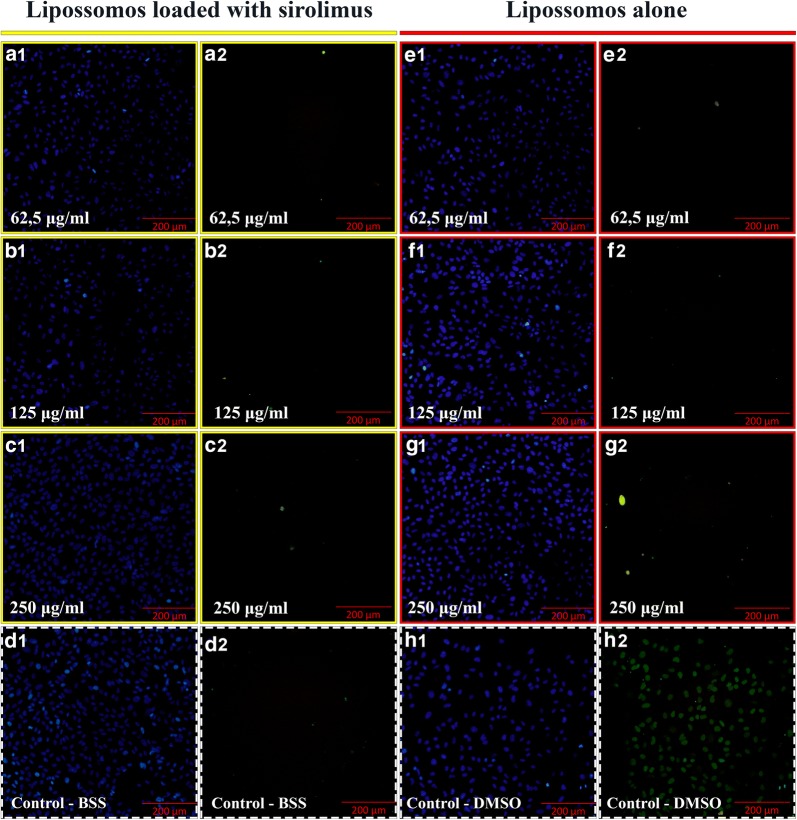
TUNEL assay with ARPE-19 cells exposed to liposome-encapsulated sirolimus (LES) (identified by yellow boxes) and liposomes alone (identified by red boxes) in different doses (62.5 µg/mL, 125 µg/mL and 250 µg/mL). The images outlined with dashed white boxes show the control, the BSS/control (**d1**–**2**) and the DMSO (dimethyl sulfoxide) positive control (**h1**–**2**). Scale bars = 200 μm

The TUNEL assay results were quantified as the proportion of apoptotic cells out of all dead cells. The percentage of apoptotic cells out of all dead cells for each group is listed in Table 3. When the frequency of dead cells was evaluated, there was no statistically significant difference in the pairwise comparisons of each groups with the control (group 7) (p = 1.0), except when comparing group 8 with the control group (p < 0.0001). There was no statistically significant difference in the pairwise comparisons between each groups (groups 1–7) (p = 1.0). The difference between each of groups 1 to 7 and group 8 was significant (all p-values < 0.0001) (Fig. 3).

**Table 3:** BSS: balanced salt solution; DMSO: dimethyl sulfoxide

Group	Medication	Apoptotic cells (%)
1	12.5 µL/mL liposome-encapsulated sirolimus	0.46 ± 0.01
2	25 µL/mL liposome-encapsulated sirolimus	0.46 ± 0.01
3	50 µL/mL liposome-encapsulated sirolimus	0.45 ± 0.01
4	12.5 µL/mL empty liposomes	0.45 ± 0.01
5	25 µL/mL empty liposomes	0.45 ± 0.01
6	50 µL/mL empty liposomes	0.45 ± 0.01
7	50 µL/mL BSS	0.45 ± 0.01
8	50 µL/mL DMSO	70.14 ± 3.05

*BSS* balanced salt solution, *DMSO* dimethyl sulfoxide

### Results of the TUNEL assay in human retinal progenitor cells

**Fig. 4:**
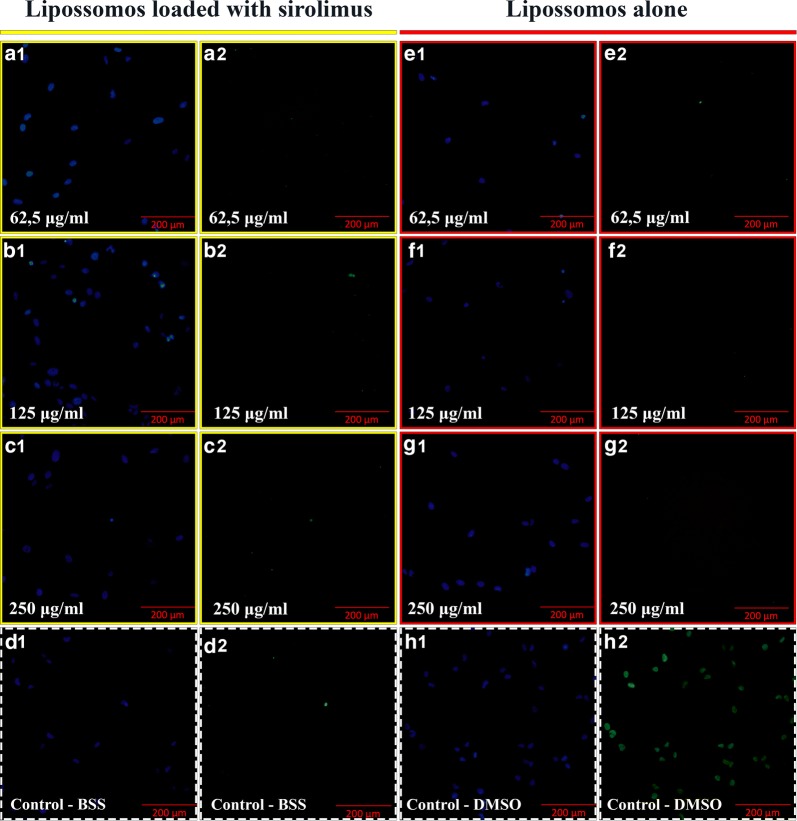
TUNEL assay with HRPC cells exposed to liposome-encapsulated sirolimus (LES) (identified by yellow boxes) and liposomes alone (identified by red boxes) in different doses (62.5 µg/mL, 125 µg/mL and 250 µg/mL). The images outlined with dashed white boxes show the control, the BSS/control (**d1**–**2**) and the DMSO (dimethyl sulfoxide) positive control (**h1**–**2**). Scale bars = 200 μm

The result was quantified in percentage of cells killed by apoptosis and presented as: group 1, 0.51% ± 0.48 cell death; group 2, 0.58% ± 0.57; group 3, 0.58% ± 0.53; group 4, 0.56% ± 0.55; group 5, 0.60% ± 0.59; group 6, 0.58% ± 0.57; group 7 (control), 0.62% ± 0.61 and group 8, 84.59% ± 0.75. When comparing groups 1 to 6 with the control group, there was no statistically significant difference (p = 1.0). The difference was significant when comparing group 8 with the control group (p < 0.0001). When comparing the groups with each other, no significant difference was observed between groups 1 to 7, but the difference was significant when comparing all groups with group 8 (p < 0.0001) (Fig. 4).

### Histopathology

**Fig. 5:**
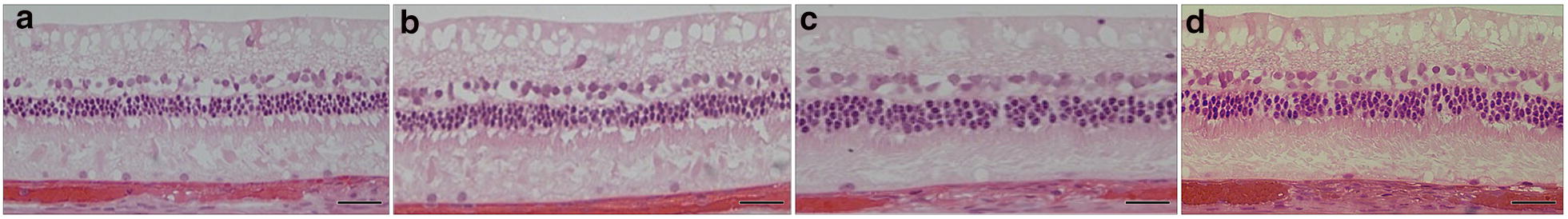
Histopathology images showing no pathological changes**: a** BSS, **b** LES 250 μg, **c** LES 500 μg, **d** liposomes alone. Scale bars = 50 μm

Pathological changes in the retinal pigment epithelium or choroidal layers were not observed in any section of the examined eyes. No opacities or inflammatory reaction were detected in the vitreous. No edema or atrophy of retinal nuclear layers were detected in any of the evaluated groups (Fig. 5).

### GFAP immunohistochemistry

**Fig. 6:**
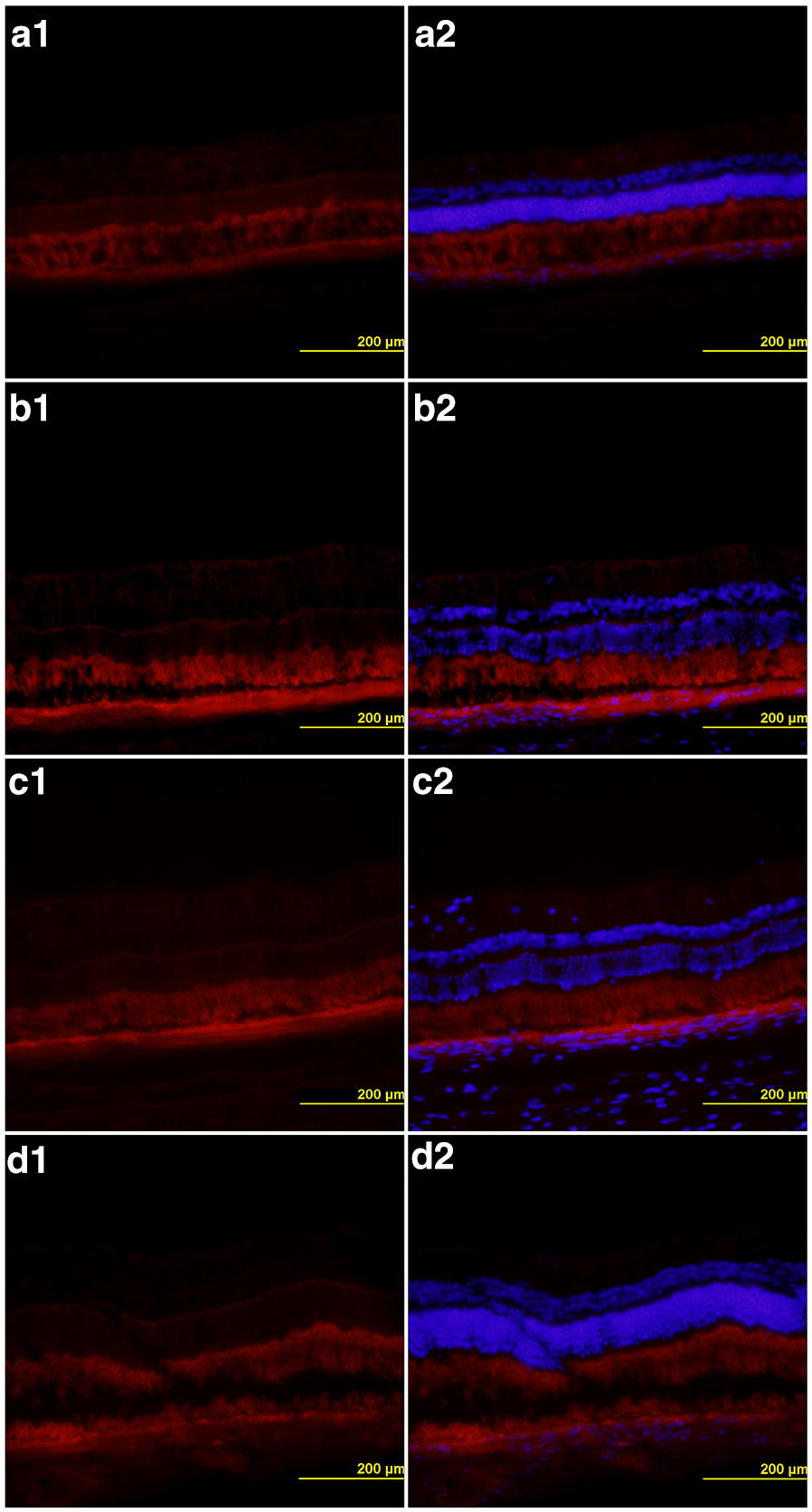
GFAP immunohistochemistry showing no activation of Müller cells: (**a1**–**2**) BSS, (**b1**–**2**) LES 250 μg, (**c1**–**2**) LES 500 μg, (**d1**–**2**) liposomes alone. Scale bars = 200 μm

None of the blades of the nine eyes examined showed activation of Müller cells (Fig. 6).

### Clinical changes

Among all the studied animals, no clinical changes including cataract, retinal detachment, vitreous inflammation, opacities or anterior chamber reactions were observed.

## Discussion

There has been considerable progress made over the last decade in the management of NIU. The SAVE study showed that an intravitreal formulation of sirolimus was well tolerated in patients with NIU over 12 months. This study also demonstrated that intravitreal sirolimus may control intraocular inflammation with better tolerability and safety profiles than systemic therapies, including immunosuppressants and corticosteroids [26, 27].

The SAKURA study, a phase III clinical trial, used intravitreal sirolimus at a dose of 440 μg, and demonstrated a significant improvement in ocular inflammation with preservation of BCVA in subjects with active posterior NIU. Subjects enrolled in the study discontinued the use of biologics and non-corticosteroid immunosuppressants before the first intravitreal sirolimus injection. The majority of the subjects did not show any ocular inflammation [28].

Our study corroborated the findings of Liu et al., who showed that sirolimus was not cytotoxic to the RPE cells using MTT tests [38]. Manzano et al. also found no histopathological changes when testing intravitreal sirolimus in rabbits using dosages between 50 and 1000 µg. To our knowledge, there have been no prior studies that have used the TUNEL assay with RPE cells along with GFAP immunohistochemistry to examine the effects of intravitreal sirolimus [39].

In plasma, sirolimus has a half-life of about 8–9 days [40]. Liposomes have been previously considered as possible means of sirolimus drug delivery, and have been shown to increase the intravitreal half-life of water-insoluble drugs such as bevacizumab and gentamicin [32, 33]. In our study, we showed that liposomes loaded with sirolimus were not toxic to ARPE-19 cells using the MTT assay and apoptosis tests. We also demonstrated that this new formulation did not present in vivo toxicity following intravitreal injections into rabbit eyes, as evaluated by histopathology and immunohistochemistry.

Further studies are needed to better assess the pharmacodynamics of liposomal forms of sirolimus.

## Conclusions

A novel formulation of liposome-encapsulated sirolimus delivered intravitreally did not cause in vitro toxicity, as evaluated by MTT and TUNEL assays, nor in vivo toxicity as evaluated by histopathology and immunohistochemistry after intravitreal administration in New Zealand rabbit eyes.

## Data Availability

The datasets used and/or analyzed during the current study are available from the corresponding author on reasonable request.

## Abbreviations

ARPE-19: acute retinal pigment epithelial-19
BCVA: best-corrected visual acuity
DNA: deoxyribonucleic acid
DMSO: dimethyl sulfoxide
EL: empty liposomes
ELISA: enzyme-linked immunosorbent assay
GFAP: glial fibrillary acidic protein
HPLC: high performance liquid chromatography
LES: liposome-encapsulated sirolimus
MTT: 3-(4,5-dimethylthiazol-2-yl)-2,5-diphenyltetrazolium bromide
NIU: non-infectious uveitis
RPE: retinal pigment epithelium
µL: microliters
